# Distracted, hyperactive, and thriving: factors supporting everyday functioning in adults with ADHD

**DOI:** 10.1186/s12888-025-06804-5

**Published:** 2025-04-23

**Authors:** Javeria Atique, Himanthri Weerawardhena, Emma A. Climie, Brandy L. Callahan

**Affiliations:** 1 Werklund School of Education, University of Calgary, Calgary, AB Canada; 2 Department of Psychology, University of Calgary, Calgary, AB Canada; 3 Hotchkiss Brain Institute, Calgary, AB Canada

**Keywords:** ADHD, Adults, Coping, Functioning, Personality, Social support, Symptoms

## Abstract

**Background:**

Using a strengths-based framework, the present study sought to identify protective personal and social characteristics associated with functional ability among adults with attention-deficit/hyperactivity disorder (ADHD).

**Methods:**

Sixty-four adults with ADHD (19–80 years old) completed self-report measures of everyday functioning, psychiatric symptoms, personality, social support, and coping strategies. Conscientious and extraverted personality traits, adaptive coping strategies, positive childhood experiences and available social support were investigated as potential protective factors supporting everyday functioning through partial correlation analyses adjusting for ADHD symptom severity and comorbid symptoms of depression and anxiety. Significant correlates were tested as modifiers of ADHD-related functional impairments in moderated regression models.

**Results:**

Multiple significant correlations were found between functional impairment and each of the personality and social characteristics of interest. Positive childhood experiences and emotion-focused coping strategies were linked to better community functioning, and positive childhood experiences were also linked to better sexual functioning. Forms of tangible, belonging and self-esteem support were associated with better functioning in social and sexual relationships, as well as with overall functional ability. None of these factors moderated the association between ADHD symptom severity and overall functioning, indicating that the functional advantages of social support were observed regardless of the severity levels of ADHD symptoms or functional impairment.

**Conclusion:**

Higher ratings of social support were linked to better functioning across many life domains independent of ADHD symptom severity, suggesting that all people with ADHD – even those with relatively low symptoms – may thrive from the development of enhanced social connections. These conclusions need to be strengthened by supportive longitudinal evidence, as it is likely that many of these associations are bi-directional.

**Supplementary Information:**

The online version contains supplementary material available at 10.1186/s12888-025-06804-5.

## Introduction

Currently, there are more adults (~ 176 million) than children (~ 130 million) living with attention-deficit/hyperactivity disorder (ADHD) worldwide (CHADD, 2023). Among these adults, 80% are impacted by their ADHD symptoms in ways that affect a variety of domains such as social functioning, educational attainment, or career success [10]. These struggles are associated with major social costs estimated at $126.2 billion USD in lost wages and healthcare overuse [24].

While existing literature on functional outcomes among individuals with ADHD has focused heavily on deficits, dysfunction, and disability, in the last 5–10 years strengths-based research on high-functioning ADHD has begun “at last entering the mainstream” [47]. This research aims to identify resources that people with ADHD might draw upon to achieve success, and highlights positive attributes of ADHD including resilience, courage, self-acceptance, motivation, and curiosity as important components of life success [68, 69]. Further identification and understanding of protective factors that can contribute to high-functioning performance in symptomatic individuals has the potential to overcome disorder stigma, promote wellbeing, and offer a new perspective that can help inform interventions [84°^86^].

For clarity, we note that there is considerable ambiguity in the scientific literature regarding the definition of ‘protection,’ which is sometimes operationalized as absence of risk. Strengths-based models of health emphasize, however, that protection and risk factors are conceptually distinct, and both can be (and often are) present simultaneously within a single individual [65]. Consistent with this definition, the present study considers protective factors as those that may offset the negative effects of risk factors, not simply representing their absence.

In the general population, several factors have been studied as potentially protective in the face of challenges. These include factors within the individual (e.g., personality traits and coping styles) and within their environment (e.g., positive social connections). In terms of personality, high extraversion has generally been associated with better adjustment to major psychological stressors such as the COVID-19 pandemic [73] and to prolonged exposure to stressful work environments [8, 49]. Extraversion may also be associated with successful functioning through its association with social competence [29]. Conscientiousness has also been consistently identified as a predictor of resilience to adversity as people encounter changes or challenges throughout their life [59, 60] and predicts successful adaptation to major stressors such as the pandemic [73]. Among other individual factors, certain coping styles have also been linked to better functional outcomes than others. Coping refers to self-regulatory mechanisms that involve using cognitive and behavioral efforts to deal with internal or external stressors [28]. Problem-focused coping, which includes active efforts to minimize the impact of a stressor (e.g., through planning, seeking instrumental support), is associated with better physical and mental functioning when faced with challenges, whereas avoidant coping (e.g., denial) generally predicts elevated distress and worse physical functioning [75]. Functional outcomes related to emotion-focused coping, which refers to managing one’s emotional responses to a stressor, are less clear because these coping strategies encompass process that are both adaptive (e.g., palliating distress through humor, seeking emotional support) and relatively more maladaptive (e.g., ruminating). Lastly, social support in the general population has been clearly tied to better functioning [46]. In childhood, positive social experiences such as positive parenting styles [78], family closeness and responsiveness [37], and parental involvement and support [67] have all been associated with subsequent positive academic, occupational, and social outcomes. In adulthood, social support is consistently linked to better physical and mental health outcomes [45] and modifies the extent to which people are disabled by symptoms of depression [79]. Overall, the literature reflects that the availability of social support can buffer the adverse effects of stress and challenges on health and everyday functioning [21].

In ADHD populations, studies of individual and environmental factors also suggest that personality, coping styles, and supportive relationships may be associated with positive functional outcomes and thus moderate the negative impacts of ADHD symptoms, although some gaps remain. In adults, self-directedness (akin to conscientiousness) favorably mediates functional outcomes such as reading ability, adjustment at work, and the development of healthy relationships [36], and is negatively associated with ADHD symptom severity [30, 31, 34, 43, 83] and with persistence of ADHD-related impairment in youth and young adults over time [1]; Millet al., 2008). Associations between ADHD and extraversion are relatively less clear. Some studies find that extraversion is significantly predictive of worse hyperactive/impulsive symptom severity [30, 42, 56] but better attentional control [32], while others find still no relationship between extraversion and cognitive functioning in ADHD [83]. Other facets of functioning (e.g., occupational or relationship successes) have not been examined in ADHD samples in relation to extraversion, to our knowledge. Regarding coping styles, adaptive strategies such as active coping, planning, and seeking support may predict successful adjustment of adults with ADHD to a major stressor (e.g., the COVID-19 pandemic: Danielsson et al., [25], however other studies find no buffering influence of adaptive coping on ADHD-related challenges [6]. Given the protective nature of adaptive coping styles in the general population [75], they are worth further exploring as a predictor of high-functioning adult ADHD. Lastly, studies of interpersonal relations in ADHD provide initial evidence that supportive mentors and partners may attenuate the level of impairment experienced by adults with ADHD.

Positive childhood experiences (PCEs), in particular, may set ADHD children up for success in adulthood: the presence of supportive childhood mentors has been identified as an important factor mitigating the manifestation and severity of emerging ADHD symptoms in young adulthood [53], and social support positively predicts the extent to which ADHD children will develop scholastic competence and social acceptance [50]. Drawing on support from parents, friends, and teachers has also been highlighted as a major factor helping college students cope with academic challenges associated with ADHD [51, 52]. Into mid- and later life, supportive spouses may play a key role in minimizing the negative impacts of ADHD (Brod, Schmitt, et al., [12]). Thus, given the broad-ranging benefits of perceived social support in mediating the difficulties associated with ADHD in childhood and emerging adulthood, there is reason to believe that it may be an influential factor in predicting successful functioning in various life domains.

The current study explores personal factors (i.e., personality traits and coping strategies) and social factors (i.e., current social support and positive early childhood experiences) that are associated with and predict functional ability in adults with ADHD. This study extends previous research in three major ways. First, the sample consists of participants with ADHD across the adult lifespan, aged 19–80 years, which broadens existing knowledge generated primarily from pediatric and emerging adult samples. Second, this study moves beyond traditional outcomes focusing on academic, occupational, and social functioning, and explores outcomes spanning across multiple life domains encompassing household, family, occupational, community, academic, health, and social functioning. Third, in contrast to prior work which has focused narrowly on risk factors, a strengths-based approach to identifying protective factors that predict high-functioning in ADHD is utilized. It is hypothesized that conscientious and extraverted personality traits, the use of adaptive coping strategies, PCEs, and available social support will be positively associated with current psychosocial and daily functioning.

## Materials and methods

### Participants

The present study was conducted as a sub-study of a larger ongoing research project at the host institution. Participants for the parent project were recruited from the local community primarily through advertisements circulated on the university campus and website, and at community events targeted at older adults. Participants from other ADHD-related research studies led by author BLC were also invited to take part in the parent project if they were eligible and had consented to future contact by the research team. Recruitment advertisements indicated that participants could take part in the parent study if they were 18 years or older, had sufficient fluency in English to complete questionnaires, had normal or corrected-to-normal hearing and vision, and had never had a stroke or dementia. A diagnosis of ADHD was not explicitly stated as an eligibility criterion, but subjects were asked two diagnostic questions upon enrollment. First, they were asked whether they had diagnosed or suspected ADHD (“Have you ever been diagnosed with, or suspected that you have/had, ADHD?”). Those who responded ‘No’ to this question were excluded from this sub-study (*n* = 19, from an initial enrolled sample of 83). Those who responded ‘Yes’ were then asked, “Was this ever formally diagnosed by a psychologist or doctor?” Those who responded ‘Yes’ to this question (n = 43) were considered to have a self-reported diagnosis of ADHD by a healthcare professional and were included in the present sub-study. Participants who had not been formally diagnosed by a healthcare professional (n = 21) were invited to complete the *Structured Clinical Interview for DSM-5* (SCID-5) administered by a trained psychometrist, and an ADHD diagnosis was confirmed in all cases. The final sample for the present study consisted of 64 individuals who met all eligibility criteria and gave written informed consent to participate. All procedures were carried out in accordance with the Declaration of Helsinki and were approved by the host institution’s Conjoint Faculties Research Ethics Board (REB22-1346). Clinical trial number: not applicable. Consent to Publish declaration: not applicable.

### Measures

Participants completed a 112-item survey inquiring about sociodemographic characteristics (age, gender, ethnicity, and education), current use of ADHD medication, psychiatric symptoms, and personal and social factors that were hypothesized to influence everyday behaviors and functional outcomes.

### ADHD symptoms

The Barkley Adult ADHD Rating Scale (BAARS-IV) is a self-report symptom rating scale consisting of 27 items measuring the frequency of inattention, hyperactivity/impulsivity, emotional dysregulation, and behavioral disinhibition [3]. It is a widely used tool in adult ADHD populations [61, 62] and was selected for use in this study as a comprehensive, brief, low-cost measure of ADHD symptom severity. Participants were instructed to respond using a four-point Likert scale ranging from 1 (never or rarely) to 4 (very often) based on their experience over the past six months. Responses are scored along subscales of inattention (with scores ranging from 4 to 36), hyperactivity (with scores ranging from 4 to 20), impulsivity (with scores ranging from 4 to 16), sluggish cognitive tempo (with scores ranging from 9 to 36) and a total ADHD symptom score (with scores ranging from 4 to 72). The total ADHD symptom score was the main measure of interest used in our analyses. Higher scores indicate worse ADHD symptom severity.

### Mood symptoms

Depressive symptoms are highly prevalent in ADHD [40, 76, 77] and can confound associations between ADHD and certain personality traits [39] or functional outcomes [9]. Therefore, a measure of depression was administered to control for the potential interaction effects with predictors of interest and influence on functional impairment. The Patient Health Questionnaire (PHQ-9) is a 9-item self-reporting questionnaire measuring the extent to which an individual has experienced depressive symptoms over the previous two weeks [44]. It is widely used in clinical and research protocols as a reliable and valid measure of depression severity because it is freely available, brief, sensitive, and specific to major depression [44]. Responses range from 0 (‘Not at all’) to 3 (‘Nearly every day’), which are summed in a final score where higher total scores are indictive of higher levels of depressive symptoms. Additionally, a measure for anxiety, a condition that frequently co-occurs with ADHD and shares similar neurobiological dysfunctions [23], was also included. The Generalized Anxiety Disorder 7-item (GAD-7; Spitzer et al., [71] scale was used as a screening tool to assess the extent to which an individual experienced anxiety symptoms over the past two weeks. Like the PHQ-9, it is widely used as a free, brief, reliable, and valid measure of anxiety severity with good sensitivity and specificity for anxiety [71]. Responses have the same range and scoring as the PHQ-9.

### Personality traits

The 60-item version of the HEXACO inventory was used to measure personality traits [2]. The HEXACO model of personality is similar in many respects to the well-known Five Factor model of personality (‘big five’) and captures all the same personality dimensions with the addition of a sixth factor. It was selected for use in this study because it is freely available from the publishers and is recognized to have better cross-cultural validity than the Five-Factor model [2]. The measure consists of six personality factors: honesty-humility (HH; refers to people who don’t feel temptation to break rules, and avoid manipulating others for personal gains), emotionality (EM; refers to a predisposition towards negative affect like fear or anxiety), extraversion (EX; refers to enjoying and feeling energized by social situations), agreeableness (AG; refers to individuals with good emotional control who compromise with others despite misgivings), conscientiousness (CO; refers to the ability to manage time and accomplish goals while maintaining discipline), and openness to experience (OE; refers to individuals who are imaginative and impressed by art and nature). Participants decided how much each item represented them on a scale from 1 (‘Strongly disagree’) to 5 (‘Strongly agree’). Each personality factor score was computed by summing all items associated with that specific factor.

### Early childhood experiences

Positive early childhood experiences were measured using the Positive Childhood Experiences Score (PCES), a 7-item scale assessing safety, sense of community/belonging, and mentorship in early life [7]. This PCEs cumulative score consists of a set of seven items taken from the well-validated 28-item Child and Youth Resilience Measure [82] and tested in a sample of > 6,000 adults at Johns Hopkins University [7]. It was selected for the present study for its brevity and clear associations with positive adulthood outcomes [7]. Each item is answered using a 5-point Likert scale; an item was determined to have been present in childhood if it received a rating of 1 (‘All the time’) or 2 (‘Most of the time’). Experiences that were rated as 3 (‘Sometimes’), 4 (‘Rarely’) or 5 (‘Never’) were considered not to have been present in childhood. A total PCES score was calculated by summing scores across all items, with higher scores indicating more positive early childhood experiences.

Because PCEs are inversely related to adverse childhood experiences (ACEs) (e.g., Bethell et al., [7], ACEs were also quantified to ensure that any effects of PCEs were not driven by the absence of ACEs. ACEs were quantified using the 11-item ACE module within the Behavioral Risk Factor Surveillance System (BRFSS) capturing household dysfunction and emotional, physical, and sexual abuse (CDC, 2019). Household dysfunction items (e.g., “Did you live with anyone who was a problem drinker or alcoholic?”) were rated as present in childhood if they were endorsed as ‘Yes.’ Abuse items (e.g., “How often did anyone at least 5 years older than you, or an adult, ever touch you sexually?”) were rated as present if they were rated as “Once” or “More than once.” Summation of the scores across all items gave a total ACEs, with higher scores indicating higher frequency of adverse experiences. Although the BRFSS relies on retrospective autobiographical recall, which may be susceptible to memory errors and biases, there is empirical support for their general validity and widespread use [35]. Of note, ethical considerations related to ACEs were carefully considered. Participants were alerted to the presence of potentially triggering questions and were provided with support resources they were encouraged to contact if they felt distressed as a result of their participation in the study.

### Social support

The 40-item Interpersonal Support Evaluation List (ISEL) assesses the availability of social support in the forms of appraisal, belonging, tangible help, and self-esteem [20]. The ISEL is widely used as a reliable, affordable index of four distinct but related functional support dimensions [13]. Appraisal support involves providing information and feedback to help a person evaluate a situation or problem. Emotional support involves providing empathy, care, and concern. Informational and tangible support involve providing information and practical assistance, respectively, to help a person deal with a problem or situation. Respondents were asked to state whether specific forms of support were available to them, on a scale from 1 (‘Definitely false’) to 4 (‘Definitely true’). A total score for each type of support was computed by summing all items associated with that type of support.

### Coping strategies

The Brief Coping Orientation to Problems Experienced inventory (Brief-COPE; Carver [14°^17^], is a measure of behavioral and cognitive strategies used by individuals to cope in stressful situations. The Brief-COPE is comprised of 28 items that are subdivided into three overarching coping styles, with eight to twelve items for each coping style. The theoretically defined overarching copying styles are emotion-focused (positive reframing, acceptance, humor, religion, using emotional support), problem-focused (active coping, planning, using instrumental support) and avoidant coping strategies (self-distraction, denial, venting, substance use, behavioral disengagement, self-blame). This measure is often used in health research as a brief alternative to lengthier instruments, and has good psychometric properties as a reliable measure of coping mechanisms [63]. Participants were asked to report on their typical use of each coping item, with no reference to specific timelines or contexts, on a scale of 1 (‘I haven’t been doing this at all’) to 4 (‘I have been doing this a lot’). Scores were summed across items within each coping style to produce three coping style scores. Because this study focus on understanding potential protective factors, only emotion-focused and problem-focused coping strategies were considered as variables of interest.

### Functional impairment

The Barkley Functional Impairment Scale (BFIS) was used to operationalize daily functioning outcomes [4]. It is a commonly used measure, not specific to ADHD, that provides a norm-based estimate of functional impairment [61]. It is brief, reliable and valid [4]. This scale has 15 items and measures the degree of impairment in various domains of functioning, including home (family and chores), work, social (strangers and friends), community activities, education, marriage/cohabiting/dating, money management, driving, sexual relations, daily responsibilities, self-care routines, health maintenance, and childrearing. Participants were instructed to estimate the extent to which they had experienced difficulties in each domain over the last six months, on a scale ranging from 0 (‘No problem’) to 8 (‘Severe problem’), with higher overall scores indicating greater functional impairment.

### Statistical analyses

The sample’s sociodemographic characteristics were summarized using descriptive statistics. To examine relationships between protective factors and functional outcomes, correlations were calculated between conscientious and extraverted personality traits, measures of social support, problem-focused and emotion-focused coping strategies, frequencies of PCEs, and BFIS subscales. These protective factors were selected for their associations with resilience in prior literature and alignment with a strengths-based definition of protection. Given the strong association between symptoms of ADHD, depression and anxiety, and functional impairment (reported below), partial correlations were computed to control for the variance they contributed to BFIS scores and provide a clearer picture of the direct effects of protective factors on functional outcomes.

Next, the personal and social factors that were significantly correlated with the BFIS total score were entered into a moderated linear regression model as modifiers of the association between ADHD symptom severity and general functional ability, also controlling for depression and anxiety. This analysis aimed to determine the extent to which protective factors could buffer the impairing impact of ADHD symptoms, as predicted by strengths-based theoretical models of protective factors.

## Results

### Sociodemographic characteristics

Sixty-four participants were included in the sample. Participants were aged 19 to 80 years (*M*_age_=24.30 years, *SD* = 15.23; Table 1). Our sample was majority female (76.6%), predominately White/European (87.5%) and most participants had completed college, trade school, or a bachelor’s degree (57.8%). Of the total sample of 64 participants, 43 self-reported a formal diagnosis of ADHD by a health professional (67.2%) and diagnostic status for the remaining 21 (33.8%) was confirmed via the SCID-5. Participants diagnosed by a health professional were not different from those diagnosed via SCID-5 in terms of ADHD symptom severity (*t*=-0.954, *p* = .350) or BFIS total score (*t*=-0.416, *p* = .679).

**Table 1:** Sample characteristics

	*N* = 64
Mean (SD) age, years	24.30 (15.23)
Gender	
Female	49 (76.6%)
Male	12 (18.8%)
Other	3 (4.7%)
Ethnicity (select all that apply)	
White/European	56 (87.5%)
South Asian (e.g., Indian, Pakistani, Bangladeshi, Nepali)	2 (3.1%)
Latino or Hispanic	2 (3.1%)
East Asian (e.g., Chinese, Japanese, Korean, Taiwanese)	3 (4.7%)
Other	2 (3.1%)
Prefer not to say	1 (1.6%)
Education completed	
Completed middle school (grade 9)	1 (1.6%)
Completed high school (grade 12 or 13)	14 (21.9%)
Completed college/trade school (2-year post-secondary degree)	15 (23.4%)
Completed a bachelor’s degree	22 (34.4%)
Completed a master’s degree	9 (14.1%)
Completed a doctoral degree (PhD or MD)	3 (4.7%)

### Correlates of functional ability

ADHD symptom severity (*r* = .640, *p* < .001) and symptoms of anxiety (*r* = .443, *p* < .001) and depression (*r* = .568, *p* < .001) were all strongly associated with overall functional impairment (Supplementary Table 1). After partialling out their variance, several significant moderate correlations were observed between various domains of functioning and the protective factors of interest (Table 2). In particular, PCEs were associated with better functional outcomes in domains of community activities (*r* = -.456, *p* = .008), sexual relations (*r* = -.472, *p* < .001) and self-care routines (*r* = -.294, *p* = .024); notably, no associations were seen between ACEs and functional outcomes, indicating that the protective impact of PCEs went beyond a mere absence of adversity. Tangible, belonging, and self-esteem forms of social support were linked to better functional outcomes in social friendships (*r* = -.324, *p* = .013; *r* = -.399, *p* = .002; *r* = -.354, *p* = .007, respectively) and sexual relations (*r* = -.399, *p* = .005; *r* = -.329, *p* = .021; *r* = -.414, *p* = .003, respectively). Self-esteem support was additionally linked to better educational outcomes (*r* = -.391, *p* = .014), belonging support was associated with social functioning with strangers (*r* = -.296, *p* = .024), and tangible support was linked to romantic relationship success (*r* = -.316, *p* = .029). Among coping styles, only emotion-focused coping was associated with functioning, though was not consistently protective: it was associated with better community functioning (*r* = -.347, *p* = .041), but worse money management (*r* = .302, *p* = .019).

**Table 2:** Partial correlation matrix, adjusted for ADHD, depression and anxiety symptom severity

	Childhood experiences		Personality traits		Social support				Coping styles	
	Positive	Adverse^a^	Extraversion	Conscientiousness	Appraisal	Tangible	Self-Esteem	Belonging	Problem-Focused	Emotion-Focused
BFIS Subscales										
Home-Family	-0.111	-0.055	0.106	0.108	-0.041	-0.104	-0.147	0.036	0.023	0.014
Home-Chores	0.014	0.005	0.158	-0.023	-0.011	-0.086	-0.108	-0.088	0.006	0.038
Work	0.050	-0.227	0.016	-0.267	-0.105	-0.099	-0.064	-0.094	0.160	-0.133
Social-Strangers	-0.115	0.122	-0.317*	-0.259	-0.163	-0.069	-0.248	-0.296*	-0.095	-0.125
Social-Friends	-0.080	0.132	-0.258	-0.116	-0.290*	-0.324*	-0.354**	-0.399**	-0.101	-0.205
Community activities	-0.456**	0.413	0.065	-0.183	-0.248	-0.321	-0.149	-0.304	-0.045	-0.347*
Education	-0.289	0.048	-0.184	-0.124	-0.147	-0.172	-0.391*	-0.119	-0.060	-0.205
Marriage/cohabiting/dating	-0.145	0.156	0.050	-0.081	-0.104	-0.316*	-0.281	-0.099	0.118	0.127
Money management	-0.001	0.109	-0.134	-0.110	-0.002	-0.118	-0.218	-0.076	-0.100	0.302*
Driving	-0.058	0.087	0.049	-0.014	0.015	0.055	-0.037	-0.030	0.024	0.090
Sexual Relations	-0.472**	0.149	-0.253	-0.238	-0.334*	-0.399**	-0.414**	-0.329*	-0.157	-0.206
Daily responsibilities	0.012	-0.122	-0.096	-0.144	-0.092	-0.153	-0.240	-0.164	0.039	0.126
Self-care routines	-0.294*	0.291	-0.154	0.116	0.000	-0.065	-0.154	-0.069	0.058	0.230
Health maintenance	-0.147	0.249	-0.176	0.167	0.017	-0.061	-0.234	-0.031	-0.064	0.183
Childrearing	0.030	0.136	0.083	-0.249	0.117	-0.159	-0.122	-0.269	-0.310	-0.006
Mean Impairment	-0.240	0.084	-0.187	-0.157	-0.197	-0.292*	-0.397**	-0.284*	-0.048	0.045

*Notes*. BFIS = Barkley Functional Impairment Scales. Values represent partial correlations, after adjusting for ADHD symptom severity and symptoms of depression and anxiety. Coefficients 0-0.19 can be interpreted as very weak associations, 0.2–0.39 as weak, 0.40–0.59 as moderate, 0.6–0.79 as strong and 0.8-1 as very strong [74]. *Correlation is significant at the 0.05 level (2-tailed). **Correlation is significant at the 0.01 level (2-tailed). ^a^Adverse childhood experiences (ACEs) are not conceptualized as protective but are included here to illustrate that significant BFIS correlations with Positive childhood experiences (PCEs) are independent from correlations with ACEs (i.e., PCEs are not simply the absence of ACEs)

Considering functional ability overall, significant correlates were tangible support, self-esteem support, and belonging support, which were all negatively correlated with overall mean impairment after accounting for ADHD symptom severity, depression, and anxiety, indicating fewer overall functioning difficulties as support in these domains increased. Moderated regression models revealed that none of the predictors significantly modified the association between ADHD symptom severity and overall functioning (Table 3). Results remained overall unchanged after correcting for age (Supplementary Tables 2 and 3).

**Table 3:** Moderated linear regression models testing the buffering effect of social support on ADHD-related functional impairment

	B	SE	β	t	*p*	95% CI
Tangible support (*R*^2^ = 0.493, *p* < .001)						
Constant	-1.366	3.121		-0.438	0.663	-7.614—4.882
ADHD symptom severity	0.152	0.063	0.750	2.396	0.020	0.025—0.279
Tangible support	0.001	0.141	0.003	0.006	0.995	-0.281—0.282
ADHD × Support (∆*R*^*2*^ = 0.001)	-0.001	0.003	− 0.200	-0.386	0.701	-0.007—0.005
Self-esteem support (*R*^2^ = 0.557, *p* < .001)						
Constant	-1.570	2.986		-0.526	0.601	-7.549—4.409
ADHD symptom severity	0.169	0.064	0.832	2.624	0.011	0.040—0.298
Self-esteem support	0.010	0.170	0.032	0.060	0.952	-0.330—0.351
ADHD × Support (∆*R*^*2*^ = 0.003)	-0.002	0.004	− 0.368	-0.652	0.517	-0.010—0.005
Belonging support (*R*^2^ = 0.482, *p* < .001)						
Constant	-1.740	3.536		-0.492	0.624	-8.817—5.337
ADHD symptom severity	0.152	0.071	0.749	2.143	0.036	0.010—0.294
Belonging support	0.025	0.177	0.090	0.140	0.889	-0.329—0.378
ADHD × Support (∆*R*^*2*^ = 0.001)	-0.001	0.004	− 0.235	-0.386	0.701	-0.009—0.006

*Notes.* ADHD = attention-deficit/hyperactivity disorder. Effect sizes are shown as β, where absolute coefficients between 0.10–0.29 can be interpreted as small, 0.30–0.49 as medium, and 0.50 or greater as large [19]

## Discussion

This study aimed to explore personal and social factors that may support everyday functioning in adults with ADHD, guided by previous findings that symptom severity is not the only predictor of an individual’s ability to contend with daily responsibilities and social relationships [31]. It was hypothesized that conscientious and extraverted personality traits, the use of adaptive coping strategies, PCEs, and available social support would be positively associated with current daily functioning. These hypotheses were only partially supported by the results: several forms of social support were moderately associated with overall functional ability, but conscientiousness, extraversion, adaptive coping styles, and PCEs were not. These findings are elaborated and discussed in detail below.

ADHD symptom severity and comorbid depressive symptoms were more strongly associated with functional abilities in this sample than were psychosocial factors. The relationship between ADHD symptoms and BFIS total impairment score was relatively stronger than the moderate correlations reported in prior work [31, 36], but ADHD symptoms did not completely predict everyday functioning. In this study, about 40% of the variance in functioning was attributable to symptom severity, indicating that it is not the only factor influencing an individual’s level of adjustment or ability. Tangible support, self-esteem support, and belonging support were also individually associated with overall functional impairment, as well as with many subdomains of functional abilities. After partialling out the variance accounting for by ADHD symptom severity, depression and anxiety, these factors explained between 8 − 16% of the variance in overall functional outcomes, with the strongest correlate being self-esteem support. The self-esteem subscale used in this study refers to the availability of people in one’s network that may bolster one’s positive identity (e.g., “There is someone who takes pride in my accomplishments”; “Most people I know think highly of me”), whereas belonging support refers to the availability of an appreciated social circle (e.g., “There are several different people I enjoy spending time with”; “When I feel lonely, there are several people I can talk to”). Relative to their peers, adults with ADHD suffer from significantly lower self-esteem [58] and are generally lonelier with fewer high-quality relationships [54, 55, 72], all which can adversely impact functioning in various life domains including mental health, social functioning and substance use [46, 58]. Consequently, bolstering self-esteem and social connection through supportive social relations may lead to improved general everyday functioning for adults with ADHD, though this conclusion would be strengthened by supportive evidence from longitudinal studies. Tangible support was also linked to functional outcomes in the present sample; this refers to the availability of practical assistance from others (e.g., “If I were sick, I could easily find someone to help me with my daily chores”). This aspect of social support was not meaningfully correlated to domains in which tangible support may be particularly beneficial (e.g., household chores, daily responsibilities, childrearing), suggesting that its link to functional outcomes may potentially reflect a general availability of positive relations in one’s social circle who simultaneously bolster one’s confidence (i.e., offering self-esteem support), provide an enjoyable social environment (i.e., offering belonging support), and are available to help with tasks if needed (i.e., offering tangible support). It is also probable that many of these associations emerged from reverse or bi-directional causality; for example, people in satisfying, high-functioning intimate or social relationships are likely to report more tangible, self-esteem, and belonging support (presumably from their partner or friends). This further highlights the need to examine these questions using longitudinal study designs to obtain clarity around the directionality of associations.

Although higher ratings of social support were linked to better functioning in areas of friendship, education, romantic and sexual relations, and overall general functioning independent of ADHD symptom severity, they did not *buffer* the negative impacts of these symptoms. Results from the moderation analysis indicated that ADHD symptom severity was associated with proportional increases in functional impairments, and this relationship was not significantly attenuated by the protective factors identified in this study. Interpreted another way, this result also means that the functional advantages of social support are observed regardless of the severity levels of ADHD symptoms or functional impairment. This suggests that all people with ADHD – even those with relatively low symptoms and impairment – may benefit from the development of enhanced social connections.

Certain results diverged from a priori hypotheses. Conscientiousness was anticipated to be functionally protective, given its previous associations with occupational and social successes in ADHD [36] and in the general population [59]. Extraversion was also hypothesized to be protective given prior positive outcomes in the face of stressors [8, 49, 73] in the general population. Neither personality trait was significantly associated with overall functioning, and only extraversion was moderately predictive of successful social functioning with strangers when considering specific domains of everyday functioning. Although these results are at odds with our a priori hypotheses, they are not altogether inconsistent with similar studies in adult ADHD. In a study of 206 adults with ADHD, self-directedness (akin to conscientiousness) predicted participants’ satisfaction at work and in primary relationships, but not educational, family or social functioning, driving, legal or criminal activity, or overall functioning [36]. The effect size for work satisfaction in that study was small (similar to the small non-significant weak association in the present study) and large for primary relationship satisfaction (whereas romantic and sexual relationship functioning in the current study were only weakly associated with conscientiousness). Thus, in contrast to research in the general population showing consistent associations between conscientiousness and positive functional outcomes across multiple domains spanning education [18], health maintenance [59], driving [48], and money management [27], results from the present sample and previous work [36] together tentatively suggest that in ADHD, personality factors may be less predictive of functional outcomes than is ADHD itself. The literature on extraversion is less consistent, and several studies in ADHD [83] and meta-analyses in the general population [18, 48] have found no association between this trait and various functional outcomes, consistent with the present results.

Further, PCEs and adaptive coping styles were not linked to overall functional outcomes, contrary to expectations. In the existing literature on PCEs [22, 33] and coping [80], their protective effects have been primarily studied on mental well-being and socioemotional functioning. It is possible that they are less predictive of the everyday tasks that often represent challenges for adults with ADHD and were tapped in the present study (e.g., staying on top of household chores, managing finances, driving), many of which are closely tied to executive functioning [5].

Lastly, although the present study was concerned principally with positive effects of protective factors, it is noteworthy that no significant correlations were observed between functional outcomes and ACEs despite a wealth of prior evidence of poor everyday functional adulthood outcomes linked to ACEs, including legal, housing, and family planning problems [62], risky driving behaviors [66], and occupational outcomes [34]. This finding is potentially explained by biases in sampling procedures (i.e., recruiting in and around an urban university community), resulting in a relatively highly educated and high socioeconomic status sample. For results to be generalized with confidence, replication to wider communities will be required in future work.

### Limitations

These results must be viewed considering some limitations. The sample was small, predominantly White, female, and well-educated, which limits generalizability to different demographics. The size of the sample limits the statistical power to detect smaller but potentially meaningful predictors of functional impairment, and it is possible that a larger sample would reveal other factors predicting functional impairment beyond depression and ADHD symptom severity. A sizeable portion of the participants in this study self-reported their diagnostic status, which is acknowledged as a potential limitation of this work. The accuracy of this information is subject to the participants’ ability to recall their diagnoses correctly and their willingness to report honestly, and there is evidence that self-reported diagnoses are not always reliable [11]. Moreover, data on participants’ date of ADHD diagnosis were not collected, which may be an important confounding factor when investigating functional ability. For example, an individual diagnosed in childhood may be relatively higher functioning because they have had more time to develop adaptive coping strategies and/or seek out accommodating environments that mitigate the functional impacts of the disorder [81]. These conclusions are also drawn with caution, as the BFIS is not designed to be used alone to measure functional impairment in all the domains captured by the questionnaire. Future studies should use medical, psychological, and clinical records alongside this scale if more generalizable claims are to be made.

Additionally, only two types of social factors (childhood experiences and current social support) and two types of personal factors (personality traits and coping styles) were explored as potential predictors of functional ability. There exist many others, which include, but are not limited to, executive functioning, family income, locus of control, physical disabilities, and comorbidities beyond mood disorders, all of which are known to impact everyday functioning [38, 57, 64]. In addition, major life transitions (e.g., retirement, relocation, death of a loved one) may have influenced the level of self-reported functional impairment [81], but these were not considered in the present study. As well, self-report measures were used to capture all the constructs, which may increase the risk of participant bias. Some participants may have felt pressured to represent themselves as highly performing due to the stigma associated with ADHD and due to social expectations that adults should be efficiently managing tasks at this stage of life. Further, there is evidence that adults with ADHD may not be reliable self-reporters: they may overestimate performance in functional activities like driving [41] and underestimate their ADHD symptom severity and level of associated impairment [26]. To address these limitations, future studies may wish to use knowledgeable co-informants alongside self-report measures, as this has been shown to increase the accuracy of symptom severity and impairment [70].

Together, this study adopted a strengths-based perspective to identify personal and social factors that can help adults with ADHD to thrive in their everyday functioning. Results suggest that social support may represent a protective factor enhancing everyday functioning in adults with ADHD, regardless of their symptom severity.

## Electronic supplementary material

Below is the link to the electronic supplementary material.

Supplementary Material 1

## Data Availability

Data will be made available upon written request to the corresponding author.
