# Supplementary material for: Distracted, hyperactive, and thriving: factors supporting everyday functioning in adults with ADHD
Source: BMC Psychiatry. 2025 Apr 23;25:418. doi: 10.1186/s12888-025-06804-5 (PMC12020315; doi:10.1186/s12888-025-06804-5)
Supplement: Supplementary file 1 — Supplementary Material 1 [file 12888_2025_6804_MOESM1_ESM.docx]

**Supplementary Table 1**

*Unadjusted correlation matrix.*

|  | Early experiences | | Personality traits | | | | | | Social support | | | | Coping strategies | | | Mood & anxiety | |
| --- | --- | --- | --- | --- | --- | --- | --- | --- | --- | --- | --- | --- | --- | --- | --- | --- | --- |
|  | Positive Childhood Experiences | Negative Childhood Experiences | Honesty-Humility | Emotionality | Extraversion | Agreeableness | Conscientiousness | Openness | Appraisal Support | Tangible Support | Self-Esteem Support | Belonging Support | Problem-Focused Coping | Emotion-Focused Coping | Avoidant Coping | Depression | Anxiety |
| BAARS-IV |  |  |  |  |  |  |  |  |  |  |  |  |  |  |  |  |  |
| ADHD Symptom Severity | -0.106 | -0.187 | -0.103 | 0.183 | -0.053 | -0.108 | -0.481^**^ | 0.014 | -0.052 | -0.231 | -0.151 | -0.371^**^ | -0.242 | 0.202 | 0.401^**^ | 0.445^**^ | 0.530^**^ |
| BFIS |  |  |  |  |  |  |  |  |  |  |  |  |  |  |  |  |  |
| Home-Family | -0.121 | -0.046 | 0.055 | 0.177 | -0.034 | -0.067 | -0.140 | 0.097 | -0.033 | -0.200 | -0.222 | -0.142 | -0.170 | 0.134 | 0.402^**^ | 0.458^**^ | 0.244 |
| Home-Chores | -0.005 | 0.049 | -0.024 | 0.245 | 0.015 | 0.010 | -0.224 | 0.134 | -0.001 | -0.170 | -0.190 | -0.216 | -0.162 | 0.143 | 0.197 | 0.479^**^ | 0.338^**^ |
| Work | 0.003 | -0.238 | 0.015 | -0.052 | -0.097 | 0.151 | -0.366^**^ | 0.255 | -0.090 | -0.190 | -0.145 | -0.217 | -0.046 | 0.000 | 0.440^**^ | 0.379^**^ | 0.111 |
| Social-Strangers | -0.096 | -0.054 | -0.247 | 0.101 | -0.333^**^ | -0.067 | -0.387^**^ | -0.020 | -0.129 | -0.131 | -0.294^*^ | -0.356^**^ | -0.194 | -0.016 | 0.371^**^ | 0.416^**^ | 0.431^**^ |
| Social-Friends | -0.064 | -0.112 | -0.136 | 0.068 | -0.282^*^ | 0.058 | -0.214 | 0.028 | -0.256^*^ | -0.332^**^ | -0.380^**^ | -0.411^**^ | -0.161 | -0.133 | 0.306^*^ | 0.297^*^ | 0.321^*^ |
| Community activities | -0.381^*^ | -0.067 | -0.235 | 0.182 | -0.018 | -0.017 | -0.327 | -0.132 | -0.200 | -0.336^*^ | -0.208 | -0.358^*^ | -0.150 | -0.205 | 0.287 | 0.415^**^ | 0.441^**^ |
| Education | -0.302^*^ | -0.030 | -0.076 | 0.084 | -0.219 | 0.135 | -0.256 | 0.191 | -0.148 | -0.245 | -0.419^**^ | -0.232 | -0.174 | -0.100 | 0.083 | 0.223 | 0.098 |
| Marriage/  cohabiting/  dating | -0.150 | 0.098 | -0.102 | -0.028 | -0.060 | 0.022 | -0.204 | 0.322^*^ | -0.088 | -0.358^**^ | -0.325^*^ | -0.196 | -0.051 | 0.202 | 0.496^**^ | 0.356^**^ | 0.121 |
| Money management | -0.047 | 0.007 | -0.172 | 0.229 | -0.180 | -0.039 | -0.349^**^ | 0.051 | -0.015 | -0.224 | -0.276^*^ | -0.262^*^ | -0.249^*^ | 0.362^**^ | 0.357^**^ | 0.411^**^ | 0.347^**^ |
| Driving | -0.098 | -0.098 | -0.251 | 0.062 | -0.004 | -0.202 | -0.255^*^ | -0.111 | -0.009 | -0.074 | -0.115 | -0.212 | -0.117 | 0.183 | 0.344^**^ | 0.279^*^ | 0.281^*^ |
| Sexual Relations | -0.364^**^ | 0.015 | -0.175 | 0.196 | -0.299^*^ | -0.078 | -0.377^**^ | -0.126 | -0.245 | -0.391^**^ | -0.428^**^ | -0.376^**^ | -0.269^*^ | -0.054 | 0.383^**^ | 0.530^**^ | 0.476^**^ |
| Daily responsibilities | -0.031 | -0.121 | -0.021 | 0.229 | -0.173 | 0.016 | -0.336^**^ | 0.014 | -0.080 | -0.244 | -0.296^*^ | -0.304^*^ | -0.149 | 0.224 | 0.373^**^ | 0.424^**^ | 0.269^*^ |
| Self-care routines | -0.268^*^ | 0.048 | 0.034 | 0.110 | -0.210 | -0.031 | -0.111 | 0.049 | 0.002 | -0.148 | -0.220 | -0.197 | -0.092 | 0.295^*^ | 0.329^**^ | 0.396^**^ | 0.322^**^ |
| Health maintenance | -0.136 | 0.048 | -0.073 | 0.254^*^ | -0.234 | -0.016 | -0.057 | 0.036 | 0.022 | -0.135 | -0.288^*^ | -0.153 | -0.188 | 0.250^*^ | 0.320^**^ | 0.412^**^ | 0.333^**^ |
| Childrearing | 0.027 | -0.152 | -0.426^*^ | -0.040 | 0.041 | 0.102 | -0.315 | 0.016 | 0.116 | -0.189 | -0.160 | -0.306 | -0.344 | 0.042 | 0.414^*^ | 0.213 | 0.229 |
| Mean Impairment Score | -0.193 | -0.061 | -0.145 | 0.186 | -0.246 | -0.039 | -0.378^**^ | 0.086 | -0.134 | -0.332^**^ | -0.401^**^ | -0.386^**^ | -0.237 | 0.175 | 0.515^**^ | 0.568^**^ | 0.443^**^ |

*. Correlation is significant at the 0.05 level (2-tailed).

**. Correlation is significant at the 0.01 level (2-tailed).

**Supplementary Table 2**

*Partial correlation matrix, adjusted for ADHD, depression and anxiety symptom severity, and age.*

|  | Childhood experiences | | Personality traits | | Social support | | | | Coping styles | |
| --- | --- | --- | --- | --- | --- | --- | --- | --- | --- | --- |
|  | Positive | Adverse^a^ | Extra-version | Conscien-tiousness | Appraisal | Tangible | Self-Esteem | Belonging | Problem-Focused | Emotion-Focused |
| BFIS Subscales |  |  |  |  |  |  |  |  |  |  |
| Home-Family | -0,111 | -0,065 | 0,102 | 0,108 | -0,027 | -0,095 | -0,139 | 0,050 | 0,018 | 0,024 |
| Home-Chores | 0,014 | 0,013 | 0,163 | -0,023 | -0,026 | -0,098 | -0,121 | -0,103 | 0,011 | 0,030 |
| Work | 0,051 | -0,250 | 0,007 | -0,268 | -0,076 | -0,078 | -0,042 | -0,070 | 0,152 | -0,115 |
| Social-Strangers | -0,115 | 0,127 | -0,316* | -0,259 | -0,178 | -0,075 | -0,259 | -0,310* | -0,093 | -0,132 |
| Social-Friends | -0,080 | 0,116 | -0,269* | -0,116 | -0,272* | -0,311* | -0,341* | -0,387** | -0,112 | -0,190 |
| Community activities | -0,462** | 0,394 | 0,050 | -0,185 | -0,207 | -0,294 | -0,115 | -0,274 | -0,063 | -0,325 |
| Education | -0,290 | 0,040 | -0,190 | -0,124 | -0,138 | -0,164 | -0,389* | -0,110 | -0,065 | -0,199 |
| Marriage/cohabiting/dating | -0,147 | 0,124 | 0,032 | -0,082 | -0,040 | -0,281 | -0,246 | -0,048 | 0,100 | 0,177 |
| Money management | -0,003 | 0,168 | -0,114 | -0,116 | -0,099 | -0,194 | -0,300* | -0,159 | -0,076 | 0,263 |
| Driving | -0,058 | 0,077 | 0,043 | -0,014 | 0,038 | 0,072 | -0,022 | -0,013 | 0,017 | 0,106 |
| Sexual Relations | -0,482** | 0,116 | -0,280 | -0,243 | -0,287* | -0,369* | -0,386** | -0,292* | -0,183 | -0,172 |
| Daily responsibilities | 0,011 | -0,107 | -0,088 | -0,145 | -0,129 | -0,180 | -0,269* | -0,196 | 0,050 | 0,109 |
| Self-care routines | -0,297* | 0,320 | -0,145 | 0,117 | -0,040 | -0,096 | -0,187 | -0,103 | 0,071 | 0,212 |
| Health maintenance | -0,150 | 0,291 | -0,163 | 0,170 | -0,044 | -0,106 | -0,286* | -0,081 | -0,047 | 0,152 |
| Childrearing | 0,030 | 0,150 | 0,090 | -0,250 | 0,100 | -0,179 | -0,141 | -0,296 | -0,305 | -0,020 |
| Mean Impairment | -0,240 | 0,090 | -0,185 | -0,157 | -0,216 | -0,306* | -0,413** | -0,301* | -0,046 | 0,040 |

*Notes*. BFIS=Barkley Functional Impairment Scales. Values represent partial correlations, after adjusting for ADHD symptom severity and symptoms of depression and anxiety. Coefficients 0-0.19 can be interpreted as very weak associations, 0.2-0.39 as weak, 0.40-0.59 as moderate, 0.6-0.79 as strong and 0.8-1 as very strong (Swinscow, 1997). *Correlation is significant at the 0.05 level (2-tailed). **Correlation is significant at the 0.01 level (2-tailed). ^a^Adverse childhood experiences (ACEs) are not conceptualized as protective but are included here to illustrate that significant BFIS correlations with Positive childhood experiences (PCEs) are independent from correlations with ACEs (i.e., PCEs are not simply the absence of ACEs).

**Supplementary Table 3**

*Age-adjusted moderated linear regression models testing the buffering effect of social support on ADHD-related functional impairment.*

|  | B | SE | β | t | *p* | 95% CI |
| --- | --- | --- | --- | --- | --- | --- |
| Tangible support (*R*^2^=.493, *p*<.001) | | | | | | |
| Constant | -1.366 | 3.121 |  | -0.438 | .663 | -7.614—4.882 |
| ADHD symptom severity | 0.152 | 0.063 | .750 | 2.396 | .020 | 0.025—0.279 |
| Tangible support | 0.001 | 0.141 | .003 | 0.006 | .995 | -0.281—0.282 |
| ADHD × Support (∆*R^2^*=.001) | -0.001 | 0.003 | -.200 | -0.386 | .701 | -0.007—0.005 |
| Age | -0.010 | 0.012 | -.083 | -0.848 | .400 | -0.034—0.014 |
| Self-esteem support (*R*^2^=.557, *p*<.001) | | | | | | |
| Constant | -1.570 | 2.986 |  | -0.526 | .601 | -7.549—4.409 |
| ADHD symptom severity | 0.169 | 0.064 | .832 | 2.624 | .011 | 0.040—0.298 |
| Self-esteem support | 0.010 | 0.170 | .032 | 0.060 | .952 | -0.330—0.351 |
| ADHD × Support (∆*R^2^*=.003) | -0.002 | 0.004 | -.368 | -0.652 | .517 | -0.010—0.005 |
| Age | -0.014 | 0.011 | -.119 | -1.294 | .201 | -0.037—0.008 |
| Belonging support (*R*^2^=.482, *p*<.001) | | | | | | |
| Constant | -1.740 | 3.536 |  | -0.492 | .624 | -8.817—5.337 |
| ADHD symptom severity | 0.152 | 0.071 | .749 | 2.143 | .036 | 0.010—0.294 |
| Belonging support | 0.025 | 0.177 | .090 | 0.140 | .889 | -0.329—0.378 |
| ADHD × Support (∆*R^2^*=.001) | -0.001 | 0.004 | -.235 | -0.386 | .701 | -0.009—0.006 |
| Age | -0.010 | 0.012 | -.083 | -0.848 | .400 | -0.034—0.014 |

*Notes.* ADHD=attention-deficit/hyperactivity disorder. Effect sizes are shown as β, where absolute coefficients between 0.10–0.29 can be interpreted as small, 0.30–0.49 as medium, and 0.50 or greater as large (Cohen, 1988).
